# Associations of Social Vulnerability Index With Pathologic Myocardial Findings at Autopsy

**DOI:** 10.3389/fcvm.2021.805278

**Published:** 2021-12-23

**Authors:** Ashwin Sunderraj, Adovich Rivera, Meghna Gaddam, Sarah Kim, Juan McCook, Janelle O'Neal, Jon Lomasney, Donald M. Lloyd-Jones, Yvonne Baumer, Tiffany M. Powell-Wiley, Matthew J. Feinstein

**Affiliations:** ^1^Clinical and Translational Immunocardiology Program, Northwestern University Feinberg School of Medicine, Chicago, IL, United States; ^2^Division of Cardiology, Department of Medicine, Northwestern University Feinberg School of Medicine, Chicago, IL, United States; ^3^Department of Pathology, Northwestern University Feinberg School of Medicine, Chicago, IL, United States; ^4^Department of Preventive Medicine, Northwestern University Feinberg School of Medicine, Chicago, IL, United States; ^5^Social Determinants of Obesity and Cardiovascular Risk Laboratory, Cardiovascular Branch, Division of Intramural Research, National Heart, Lung, and Blood Institute, Bethesda, MD, United States; ^6^Intramural Research Program, National Institute on Minority Health and Health Disparities, Bethesda, MD, United States

**Keywords:** social vulnerability, cardiac death, myocardial fibrosis, heart failure, pathology

## Abstract

**Background:** Social vulnerability is an important determinant of cardiovascular health. Prior investigations have shown strong associations of social determinants of health with cardiovascular risk factors, imaging findings, and clinical events. However, limited data exist regarding the potential role of social vulnerability and related physiologic stressors on tissue-level pathology.

**Methods:** We analyzed clinical data and linked autopsy reports from 853 decedent individuals who underwent autopsy from 4/6/2002 to 4/1/2021 at a large urban medical center. The mean age at death was 62.9 (SD = 15.6) and 49% of decedent individuals were men. The primary exposure was census-tract level composite social vulnerability index based on the Centers for Disease Control and Prevention Social Vulnerability Index (SVI). Individuals were geocoded to census tracts and assigned SVI accordingly. Four myocardial tissue-level outcomes from autopsy were recorded as present or absent: any coronary atherosclerosis, severe/obstructive coronary atherosclerosis, myocardial fibrosis, and/or myopericardial inflammation. Multivariable-adjusted logistic regression models were constructed with SVI as the primary exposure and covariates including age, sex, race, body mass index (BMI), diabetes, and hypertension. Additional analyses were performed stratified by clinical diagnoses of heart failure (HF) and coronary artery disease (CAD).

**Results:** In the overall cohort, SVI was not associated with outcomes on cardiac pathology in multivariable-adjusted models. However, in stratified multivariable-adjusted analyses, higher SVI (higher social vulnerability) was associated with a higher odds of myocardial fibrosis among individuals without clinical diagnoses of HF.

**Conclusions:** Higher indices of social vulnerability are associated with a higher odds of myocardial fibrosis at autopsy among individuals without known clinical diagnoses of HF. Potential pathophysiological mechanisms and implications for prevention/treatment of myocardial dysfunction require further study.

## Introduction

Social determinants of health span a variety of individual characteristics as well as material circumstances and have a significant impact on cardiovascular health and disease. The American Heart Association considers education, income, employment status, social networks, and access to medical care or other health-promoting resources (often associated with disparate access by race and ethnicity) as major determinants of cardiovascular risk and health (1). Disparities across these categories are well described (2), with Black Americans two to three times as likely as White Americans to die of preventable heart disease. Despite overall declines in mortality (3), an >19,000 excess Black deaths from heart disease across the 50 largest U.S cities occur annually (4). These disparities are intersectional in nature, with higher levels of educational attainment among Hispanic and Black Americans showing diminished decreases in heart disease mortality relative to non-Hispanic Whites (5). Additionally, women have previously been found to have a lower prevalence of CVD risk factors than men in a major metropolitan area, but this difference was largely restricted to non-Latino white women, with women in other groups having a higher prevalence of these same risk factors – further illustrating the intersectional nature of cardiovascular disease burden (6). Socioeconomic circumstances, such as access to housing (7) and one's neighborhood of residence (8–10), have also been tied to adverse cardiovascular outcomes.

Social conditions have previously been described as influencing physical health outcomes through the process of embodiment (11). While the associations between social determinants of health and clinical outcomes are well established, less granularity exists regarding the relationship between these factors and tissue-level myocardial pathology. These manifestations, such as myocardial fibrosis, contribute to both systolic and diastolic dysfunction as well as heart failure (HF) and are induced by a complex interplay of physiologic stressors. Myocardial fibrosis is involved in the pathogenesis of natural aging, as well as hypertension, heart failure, and aortic stenosis (12). Myocardial fibrosis has also been described as inducing a “self-organizing criticality,” in which complex adaptations to progressive stress abruptly end in sudden cardiac death, in the absence of any change in the underlying pathology (13). The drivers of these processes, and potential contribution of social determinants of health, are incompletely defined.

The Centers for Disease Control and Prevention have established a Social Vulnerability Index (SVI) to rank social vulnerability at the census-tract level, measured as a composite of 15 social factors, including minority status, disability status, unemployment, and educational attainment. SVI has previously been used in investigation disproportionate impacts of COVID-19 on vulnerable communities (14), emergency medical service utilization (15), and likelihood of undergoing emergent as opposed to elective surgical procedures (16). Therefore, in this study, we investigated whether SVI was associated with tissue-level myocardial pathology on autopsy.

## Materials and Methods

### Study Population

This is a retrospective analysis of a cohort of 853 decedent individuals who underwent autopsy from 4/6/2002 to 4/1/2021 at Northwestern Memorial Hospital, a single urban center located in Chicago, IL, had clinical data available for analysis, and who resided in IL according to their chart-documented addresses. Individuals were geocoded to census tracts based on addresses and assigned an SVI value, as discussed next. Patients were excluded if their address was located outside the state of Illinois because the vast majority of individuals lived in Illinois and considering a single state ensured harmonization of our scaled SVI measure.

### Social Vulnerability Index

This investigation uses the 2018 CDC Social Vulnerability Index, specifically the overall tract summary ranking variable. Census tracts within the state of Illinois were given an SVI value ranging from 0.000 to 1.000, with 1.000 representing the 100^th^ percentile for extreme social vulnerability and 0.000 representing the 0^th^ percentile for the lowest level social vulnerability, ranked against one another.

### Statistical Analysis

The 2018 CDC SVI was the primary exposure in this analysis. Outcomes of interest related to the presence or absence of cardiac autopsy findings, including any atherosclerosis, severe atherosclerosis, myocardial fibrosis, and/or myopericardial inflammation. These outcomes were determined on cardiac autopsy by a board-certified pathologist.

We used logistic regressions with sequential adjustment to evaluate associations of SVI with pathologic outcomes. These included investigating univariate associations between SVI and cardiac autopsy outcomes (Model 1), second, multivariable-adjusted associations between SVI and cardiac autopsy outcomes adjusting for age, sex, race/ethnicity (Model 2), and multivariable associations adjusting for age, sex, race/ethnicity, BMI, diabetes status, and hypertension status (Model 3). Race and ethnicity in this study was abstracted from medical records, and categorized as non-Hispanic white, non-Hispanic black, or Asian, Hispanic or Other. The Asian, Hispanic, or Other group was formed due to the relatively small sample size of these individual categories, which were originally found in the clinical charts. Given the known effects of coronary artery disease (CAD) and myocardial infarction (MI) as well as HF on myocardial pathology, we performed pre-specified secondary analyses of the above models stratified by clinical diagnoses of CAD and HF. Coronary artery disease and heart failure were defined using administrative code-based definitions as described previously in analyses of electronic health record data in the same healthcare system (17). We additionally stratified our population by the presence or absence of clinical myocardial infarction, as defined by ICD code on chart review, and replicated these sequential analyses on the association of SVI with cardiac pathological traits on autopsy. In all models, we report odds ratio (OR), 95% confidence intervals, and *p* values atalpha = 0.05.

All analyses were performed using R/RStudio version 1.4.1103.

### Pathologic Cardiac Outcomes

Four pathologic cardiac outcomes were identified at autopsy by a trained pathologist: myocardial fibrosis, any coronary atherosclerosis, severe coronary atherosclerosis, and myocarditis and/or pericardial inflammation. Myocardial fibrosis was defined pathologically and, accordingly, includes myocardial fibrosis that may have resulted from ischemic and non-ischemic causes. Myocardial fibrosis was defined dichotomously as the presence or absence of any cardiac fibrosis on autopsy. Severe coronary atherosclerosis was defined as >50% obstruction of one or more major epicardial coronary arteries on autopsy, while any coronary atherosclerosis was defined as the presence of any atherosclerosis of any epicardial coronary artery on autopsy. Myocarditis and/or Pericardial Inflammation was defined as the presence of inflammatory cell infiltrate.

### Ethical Approval Statement

This study was approved by the Northwestern University Institutional Review Board (STU 00202918).

## Results

After linkage of clinical charts and autopsy reports, *N* = 1,296 decedent individuals were identified. After removing patients with invalid address information and incomplete clinical or autopsy data, a cohort of *N* = 853 decedent individuals remained and used for these analyses.

In this cohort, mean SVI was 0.49 (SD 0.29), as expected from a percentile-based variable. Individuals were 62.90 years old at death on average (SD = 15.61); Non-Hispanic Whites represented the majority of the population (41.5%), with Non-Hispanic Blacks representing approximately one-third (33.2%), and Asian, Hispanic, and other groups representing 25.3% of the population respectively (Table 1). The majority of participants did not have diabetes (59.4%), did not have hypertension (63.3%), were without HF (56.0%) and without CAD (54.7%) (Table 1). However, the majority of subpopulations with adverse myocardial pathology at autopsy – severe coronary atherosclerosis, myocardial fibrosis, and myocarditis and/or pericardial inflammation – had clinical diagnoses of heart failure (55.9, 58.0, 58.0%) and CAD (71.5, 59.2, 54.5) respectively (Table 1). The majority of patients in the subpopulation with any atherosclerosis had CAD (52.3%) (Table 1).

**Table 1:** Overview of clinical and demographic differences across autopsy-based measurement categories.

	**Overall**	**Coronary atherosclerosis (Any)**	**Coronary atherosclerosis (Severe)**	**Myocardial fibrosis**	**Myocarditis and/or pericardial inflammation**
*N*	853	639	256	417	143
Social vulnerability index [mean (SD)]	0.49 (0.29)	0.49 (0.29)	0.49 (0.29)	0.51 (0.29)	0.49 (0.28)
**Age [mean (SD)]**	62.90 (15.61)	66.98 (13.44)	68.67 (13.17)	65.25 (14.68)	60.2 (15.92)
**Race [*****N*** **(%)]**					
Non-Hispanic white	354 (41.5)	279 (43.7)	115 (44.9)	159 (38.1)	59 (41.3)
Asian, Hispanic, and other	216 (25.3)	155 (24.3)	58 (22.7)	104 (24.9)	40 (28.0)
Non-Hispanic black	283 (33.2)	205 (32.1)	83 (32.4)	154 (36.9)	44 (30.8)
**Diabetes status [*****N*** **(%)]**					
Yes	346 (40.6)	288 (45.1)	127 (49.6)	195 (46.8)	64 (44.8)
No	507 (59.4)	351 (54.9)	129 (50.4)	222 (53.2)	79 (55.2)
**Hypertension status [*****N*** **(%)]**					
Yes	313 (36.7)	262 (41.0)	114 (44.5)	180 (43.2)	54 (37.8)
No	540 (63.3)	377 (59.0)	143 (55.5)	237 (56.8)	89 (62.2)
BMI [mean (SD)]	28.26 (7.63)	28.35 (7.52)	28.40 (7.49)	28.66 (7.82)	28.90 (7.72)
**Heart failure status [(*****N*** **(%)]**					
Yes	375 (44.0)	308 (48.2)	143 (55.9)	242 (58.0)	83 (58.0)
No	478 (56.0)	331 (51.8)	113 (44.1)	175 (42.0)	60 (42.0)
**Coronary artery disease status [*****N*** **(%)]**					
Yes	386 (45.3)	334 (52.3)	183 (71.5)	247 (59.2)	78 (54.5)
No	467 (54.7)	305 (47.7)	73 (28.5)	170 (40.8)	65 (45.5)

There was no significant association between SVI and any pathologic cardiac outcome in the overall cohort (Table 2). Statistically significant associations were identified for male sex and age, but not race, with several pathologic cardiac outcomes (Table 2). However, no statistically significant association between race and any pathologic cardiac outcome was observed (Table 2). Both diabetes status and hypertension status were associated with increased odds ratios for several pathologic cardiac outcomes (Table 2).

**Table 2:** Associations of social vulnerability index with cardiac pathology at autopsy.

	**Coronary atherosclerosis (Any)**		**Coronary atherosclerosis (Severe)**		**Myocardial fibrosis**		**Myocarditis and/or pericardial inflammation**	
**Exposure**	**OR 95% CI**	***P* value**	**OR 95% CI**	***P* value**	**OR 95% CI**	***P* value**	**OR 95% CI**	***P* value**
Social vulnerability index (Model 1)	0.71 [0.42, 1.21]	*p* = 0.21	0.97 [0.59, 1.61]	*p* = 0.91	1.57 [0.99, 2.50]	*p* = 0.06	0.95 [0.51, 1.76]	*p* = 0.87
Social vulnerability index (Model 2)	1.60 [0.82, 3.13]	*p* = 0.17	1.47 [0.81, 2.66]	*p* = 0.21	1.56 [0.91, 2.66]	*p* = 0.10	0.90 [0.45, 1.81]	*p* = 0.78
Social vulnerability index (Model 3)	1.39 [0.70, 2.73]	*p* = 0.35	1.32 [0.73, 2.40]	*p* = 0.36	1.45 [0.85, 2.46]	*p* = 0.17	0.94 [0.47, 1.87]	*p* = 0.86
**Race (ref = White)**								
Asian/Hispanic/Other	0.72 [0.45, 1.15]	*p* = 0.17	0.72 [0.48, 1.09]	*p* = 0.12	1.06 [0.74, 1.53]	*p* = 0.75	1.06 [0.67, 1.69]	*p* = 0.80
Black	0.73 [0.46, 1.16]	*p* = 0.18	0.82 [0.55, 1.24]	*p* = 0.35	1.34 [0.93, 1.92]	*p* = 0.12	0.90 [0.56, 1.46]	*p* = 0.68
**Sex (ref = Female)**								
Male	1.65^**^ [1.14, 2.38]	*p* = 0.01	1.52^**^ [1.12, 2.07]	*p* = 0.01	1.54^**^ [1.16, 2.04]	*p* = 0.00	1.27 [0.88, 1.83]	*p* = 0.20
Age	1.08^^**^*^ [1.06, 1.09]	*p* = 0.00	1.04^^**^*^ [1.02, 1.05]	*p* = 0.00	1.02^^**^*^ [1.01, 1.03]	*p* = 0.00	0.98^*^ [0.97, 1.00]	*p* = 0.01
Body mass index	1.00 [1.00, 1.00]	*p* = 0.45	1.00 [1.00, 1.00]	*p* = 0.73	1.00 [1.00, 1.00]	*p* = 0.59	1.00 [0.99, 1.01]	*p* = 0.79
**Diabetes status (ref = No)**								
Yes	2.28^^**^*^ [1.54, 3.37]	*p* = 0.00	1.68^**^ [1.23, 2.31]	*p* = 0.00	1.44^*^ [1.08, 1.92]	*p* = 0.01	1.26 [0.87, 1.83]	*p* = 0.22
**Hypertension status (ref = No)**								
Yes	1.40 [0.93, 2.11]	*p* = 0.10	1.23 [0.89, 1.69]	*p* = 0.22	1.39^*^ [1.03, 1.87]	*p* = 0.03	1.17 [0.79, 1.74]	*p* = 0.42
*N*	871		871		871		871	

*Model 1, Unadjusted; Model 2, Adjusted for age, sex, and race; Model 3, Adjusted for age, sex, race, body mass index, diabetes, and hypertension status*.

*** *p < 0.001*;

** *p < 0.01*;

* *p < 0.05*.

**Figure 1:**
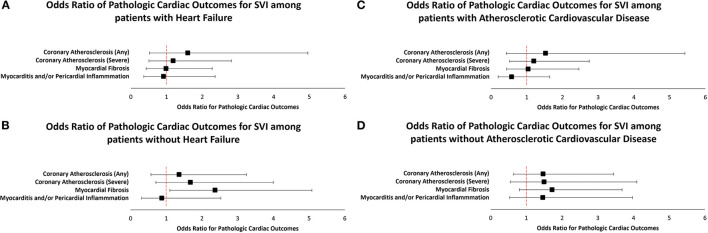
Multivariable adjusted odds ratios for pathologic cardiac outcomes for SVI among patients stratified by heart failure **(A,B)** and coronary artery disease **(C,D)** status.

Among patients with HF, no statistically significant associations between SVI and pathologic cardiac outcomes were found (Table 3A). Among patients without HF, a statistically significant association was observed between higher SVI and myocardial fibrosis (Odds Ratio (OR) = 2.37, 95% Confidence Interval (CI) 1.10–5.08, p = 0.03; Figure 1). Because the OR of 2.37 (or an absolute increase of 137%) is for the highest (1.00) vs. lowest (0.00) SVI, every 10% increase in SVI was associated with a 13.7% (137%/10 = 13.7%) increase in odds of having myocardial fibrosis at autopsy. This association was observed in the final multivariable-adjusted model and was also noted in the demographically adjusted models, but not the univariate model (Table 3B). No significant associations of SVI and other pathologic cardiac outcomes were detected (Table 3B).

**Table 3A:** Associations of social vulnerability index with cardiac pathology at autopsy among individuals with clinical diagnoses of heart failure.

	**Coronary atherosclerosis (Any)**		**Coronary atherosclerosis (Severe)**		**Myocardial fibrosis**		**Myocarditis and/or pericardial inflammation**	
**Exposure**	**OR 95% CI**	***P* value**	**OR 95% CI**	***P* value**	**OR 95% CI**	***P* value**	**OR 95% CI**	***P* value**
Social vulnerability index (Model 1)	0.83 [0.33, 2.04]	*p* = 0.68	1.04 [0.51, 2.13]	*p* = 0.90	1.31 [0.63, 2.70]	*p* = 0.47	0.95 [0.41, 2.20]	*p* = 0.91
Social vulnerability index (Model 2)	1.72 [0.57, 5.23]	*p* = 0.34	1.21 [0.52, 2.82]	*p* = 0.66	1.00 [0.44, 2.28]	*p* = 0.99	0.94 [0.36, 2.40]	*p* = 0.89
Social vulnerability index (Model 3)	1.60 [0.52, 4.96]	*p* = 0.42	1.19 [0.51, 2.82]	*p* = 0.69	0.99 [0.43, 2.28]	*p* = 0.98	0.92 [0.36, 2.36]	*p* = 0.86
**Race (ref = White)**								
Asian/Hispanic/Other	0.50 [0.22, 1.14]	*p* = 0.10	0.74 [0.40, 1.36]	*p* = 0.33	1.18 [0.66, 2.11]	*p* = 0.57	1.22 [0.64, 2.32]	*p* = 0.55
Black	0.47 [0.20, 1.09]	*p* = 0.08	1.01 [0.55, 1.82]	*p* = 0.99	1.64 [0.91, 2.96]	*p* = 0.10	0.85 [0.43, 1.68]	*p* = 0.64
**Sex (ref = Female)**								
Male	1.43 [0.76, 2.69]	*p* = 0.27	1.67^*^ [1.05, 2.65]	*p* = 0.03	1.70^*^ [1.08, 2.68]	*p* = 0.02	1.17 [0.70, 1.95]	*p* = 0.56
Age	1.08^***^ [1.06, 1.10]	*p* = 0.00	1.04^***^ [1.02, 1.05]	*p* = 0.00	1.01 [0.99, 1.02]	*p* = 0.24	0.98^*^ [0.96, 1.00]	*p* = 0.01
Body mass index	1.02 [0.98, 1.06]	*p* = 0.35	1.01 [0.99, 1.04]	*p* = 0.33	1.01[0.98, 1.04]	*p* = 0.55	1.02 [0.98, 1.05]	*p* = 0.34
**Diabetes status (ref = No)**								
Yes	1.80 [0.94, 3.43]	*p* = 0.08	1.86^**^ [1.17, 2.95]	*p* = 0.01	1.68^*^ [1.06, 2.66]	*p* = 0.03	1.13 [0.67, 1.90]	*p* = 0.64
**Hypertension status (ref = No)**								
Yes	1.40 [0.72, 2.71]	*p* = 0.32	1.09 [0.69, 1.72]	*p* = 0.72	0.85 [0.53, 1.34]	*p* = 0.48	1.01 [0.60, 1.71]	*p* = 0.97
N	375		375		375		375	

*Model 1, Unadjusted; Model 2, Adjusted for age, sex, and race; Model 3, Adjusted for age, sex, race, body mass index, diabetes, and hypertension status*.

*** *p < 0.001*;

** *p < 0.01*;

* *p < 0.05*.

**Table 3B:** Associations of social vulnerability index with cardiac pathology at autopsy among individuals without clinical diagnoses of heart failure.

	**Coronary atherosclerosis (Any)**		**Coronary atherosclerosis (Severe)**		**Myocardial fibrosis**		**Myocarditis and/or pericardial inflammation**	
**Exposure**	**OR 95% CI**	***P* value**	**OR 95% CI**	***P* value**	**OR 95% CI**	***P* value**	**OR 95% CI**	***P* value**
Social vulnerability index (Model 1)	0.63 [0.32, 1.23]	*p* = 0.18	0.87 [0.42, 1.80]	*p* = 0.70	1.85 [0.97, 3.54]	*p* = 0.06	0.91 [0.36, 2.31]	*p* = 0.84
Social vulnerability index (Model 2)	1.57 [0.67, 3.67]	*p* = 0.30	1.71 [0.73, 4.05]	*p* = 0.22	2.39^*^ [1.12, 5.07]	*p* = 0.02	0.90 [0.32, 2.56]	*p* = 0.84
Social vulnerability index (Model 3)	1.36 [0.57, 3.25]	*p* = 0.49	1.68 [0.71, 4.00]	*p* = 0.24	2.37^*^ [1.10, 5.08]	*p* = 0.03	0.88 [0.31, 2.53]	*p* = 0.82
**Race (ref = White)**								
Asian/Hispanic/Other	0.83 [0.46, 1.49]	*p* = 0.53	0.74 [0.42, 1.32]	*p* = 0.31	1.01 [0.60, 1.68]	*p* = 0.98	0.86 [0.42, 1.76]	*p* = 0.68
Black	0.79 [0.44, 1.42]	*p* = 0.44	0.66 [0.36, 1.18]	*p* = 0.16	1.18 [0.71, 1.95]	*p* = 0.53	0.92 [0.45, 1.87]	*p* = 0.81
**Sex (ref = Female)**								
Male	1.74^*^ [1.09, 2.75]	*p* = 0.02	1.45 [0.93, 2.27]	*p* = 0.10	1.44 [0.97, 2.13]	*p* = 0.07	1.38 [0.79, 2.42]	*p* = 0.25
Age	1.08^***^ [1.06, 1.10]	*p* = 0.00	1.04^***^ [1.02, 1.06]	*p* = 0.00	1.03^***^ [1.01, 1.04]	*p* = 0.00	0.99 [0.97, 1.01]	*p* = 0.20
Body mass index	1.01 [0.98, 1.05]	*p* = 0.42	1.01 [0.98, 1.04]	*p* = 0.51	1.02 [1.00, 1.05]	*p* = 0.09	1.00 [0.96, 1.04]	*p* = 0.91
**Diabetes status (ref = No)**								
Yes	2.29^**^ [1.37, 3.83]	*p* = 0.00	1.25 [0.78, 2.02]	*p* = 0.35	0.99 [0.65, 1.51]	*p* = 0.97	1.14 [0.63, 2.05]	*p* = 0.66
**Hypertension status (ref = No)**								
Yes	1.31 [0.76, 2.29]	*p* = 0.33	1.13 [0.69, 1.85]	*p* = 0.63	1.53 [0.99, 2.37]	*p* = 0.06	1.04 [0.55, 1.98]	*p* = 0.91
N	478		478		478		478	

*Model 1, Unadjusted; Model 2, Adjusted for age, sex, and race; Model 3, Adjusted for age, sex, race, body mass index, diabetes, and hypertension status*.

*** *p < 0.001*;

** *p < 0.01*;

* *p < 0.05*.

Among patients with CAD, no statistically significant associations between SVI and pathologic cardiac outcomes were found (Table 3C). Additionally, among patients without CAD, no statistically significant associations between SVI and pathologic cardiac outcomes were observed (Table 3D; Figure 1).

**Table 3C:** Associations of social vulnerability index with cardiac pathology at autopsy among individuals with clinical diagnoses of coronary artery disease.

	**Coronary atherosclerosis (Any)**		**Coronary atherosclerosis (Severe)**		**Myocardial fibrosis**		**Myocarditis and/or pericardial inflammation**	
**Exposure**	**OR 95% CI**	***P* value**	**OR 95% CI**	***P* value**	**OR 95% CI**	***P* value**	**OR 95% CI**	***P* value**
Social vulnerability index (Model 1)	0.67 [0.24, 1.85]	*p* = 0.44	0.83 [0.42, 1.66]	*p* = 0.60	1.40 [0.68, 2.88]	*p* = 0.36	0.64 [0.27, 1.52]	*p* = 0.32
Social vulnerability index (Model 1)	1.45 [0.42, 4.98]	*p* = 0.55	1.15 [0.51, 2.63]	*p* = 0.74	0.98 [0.42, 2.29]	*p* = 0.96	0.51 [0.18, 1.41]	*p* = 0.19
Social vulnerability index (Model 3)	1.53 [0.43, 5.43]	*p* = 0.51	1.20 [0.52, 2.75]	*p* = 0.67	1.04 [0.44, 2.46]	*p* = 0.93	0.58 [0.20, 1.64]	*p* = 0.30
**Race (ref = White)**								
Asian/Hispanic/Other	0.42 [0.17, 1.02]	*p* = 0.06	0.65 [0.37, 1.15]	*p* = 0.14	1.54 [0.85, 2.77]	*p* = 0.15	1.29 [0.64, 2.58]	*p* = 0.48
Black	0.46 [0.19, 1.13]	*p* = 0.09	0.80 [0.46, 1.40]	*p* = 0.43	1.60 [0.89, 2.86]	*p* = 0.12	1.10 [0.53, 2.26]	*p* = 0.81
**Sex (ref = Female)**								
Male	1.85 [0.97, 3.53]	*p* = 0.06	1.49 [0.98, 2.27]	*p* = 0.06	1.42 [0.91, 2.20]	*p* = 0.12	1.06 [0.62, 1.80]	*p* = 0.83
Age	1.06^***^ [1.03, 1.08]	*p* = 0.00	1.02^**^ [1.01, 1.04]	*p* = 0.01	1.00 [0.99, 1.02]	*p* = 0.63	0.98^*^ [0.96, 1.00]	*p* = 0.02
Body mass index	1.03 [0.98, 1.07]	*p* = 0.22	1.01 [0.99, 1.04]	*p* = 0.37	1.00 [0.97, 1.03]	*p* = 0.83	1.02 [0.99, 1.06]	*p* = 0.21
**Diabetes status (ref = No)**								
Yes	1.83 [0.95, 3.53]	*p* = 0.07	1.27 [0.82, 1.95]	*p* = 0.28	1.12 [0.72, 1.75]	*p* = 0.61	1.12 [0.66, 1.92]	*p* = 0.67
**Hypertension status (ref = No)**								
Yes	1.55 [0.80, 3.01]	*p* = 0.20	0.92 [0.60, 1.40]	*p* = 0.68	0.98 [0.63, 1.53]	*p* = 0.94	0.63 [0.37, 1.07]	*p* = 0.09
**Heart failure status (ref = No)**								
Yes	1.09 [0.55, 2.13]	*p* = 0.81	0.93 [0.60, 1.45]	*p* = 0.75	1.91^**^ [1.22, 3.00]	*p* = 0.00	2.29^**^ [1.24, 4.24]	*p* = 0.01
N	386		386		386		386	

*Model 1, Unadjusted; Model 2, Adjusted for age, sex, and race; Model 3, Adjusted for age, sex, race, body mass index, diabetes, and hypertension*.

*** *p < 0.001*;

** *p < 0.01*;

* *p < 0.05*.

**Table 3D:** Associations of social vulnerability index with cardiac pathology at autopsy among individuals without clinical diagnoses of coronary artery disease.

	**Coronary atherosclerosis (Any)**		**Coronary atherosclerosis (Severe)**		**Myocardial fibrosis**		**Myocarditis and/or pericardial inflammation**	
**Exposure**	**OR 95% CI**	***P* value**	**OR 95% CI**	***P* value**	**OR 95% CI**	***P* value**	**OR 95% CI**	***P* value**
Social vulnerability index (Model 1)	0.62 [0.32, 1.20]	*p* = 0.16	0.89 [0.38, 2.10]	*p* = 0.79	1.58 [0.82, 3.01]	*p* = 0.17	1.35 [0.55, 3.31]	*p* = 0.51
Social vulnerability index (Model 2)	1.67 [0.73, 3.80]	*p* = 0.23	1.66 [0.61, 4.49]	*p* = 0.32	1.95 [0.94, 4.04]	*p* = 0.07	1.42 [0.53, 3.81]	*p* = 0.48
Social vulnerability index (Model 3)	1.47 [0.63, 3.44]	*p* = 0.38	1.50 [0.55, 4.09]	*p* = 0.43	1.72 [0.80, 3.68]	*p* = 0.16	1.46 [0.53, 3.97]	*p* = 0.46
**Race (ref = White)**								
Asian/Hispanic/Other	0.90 [0.50, 1.61]	*p* = 0.72	0.85 [0.43, 1.65]	*p* = 0.63	0.82 [0.49, 1.39]	*p* = 0.47	0.84 [0.43, 1.66]	*p* = 0.62
Black	0.78 [0.44, 1.39]	*p* = 0.40	0.76 [0.38, 1.51]	*p* = 0.43	1.26 [0.75, 2.10]	*p* = 0.38	0.68 [0.34, 1.38]	*p* = 0.29
**Sex (ref = Female)**								
Male	1.42 [0.90, 2.26]	*p* = 0.13	1.24 [0.73, 2.12]	*p* = 0.42	1.52^*^ [1.01, 2.29]	*p* = 0.05	1.47 [0.85, 2.52]	*p* = 0.17
Age	1.08^***^ [1.07, 1.10]	*p* = 0.00	1.04^***^ [1.02, 1.06]	*p* = 0.00	1.02^**^ [1.01, 1.03]	*p* = 0.00	0.98 [0.97, 1.00]	*p* = 0.05
Body mass index	1.01 [0.98, 1.04]	*p* = 0.45	1.01 [0.98, 1.05]	*p* = 0.55	1.03^*^ [1.00, 1.06]	*p* = 0.02	0.99 [0.96, 1.03]	*p* = 0.69
**Diabetes status (ref = No)**								
Yes	2.05^**^ [1.21, 3.47]	*p* = 0.01	1.38 [0.77, 2.46]	*p* = 0.27	1.21 [0.77, 1.90]	*p* = 0.40	0.93[0.51, 1.72]	*p* = 0.83
**Hypertension status (ref = No)**								
Yes	1.04 [0.59, 1.83]	*p* = 0.89	1.02 [0.56, 1.85]	*p* = 0.96	1.17 [0.73, 1.86]	*p* = 0.52	1.83 [0.99, 3.38]	*p* = 0.05
**Heart failure status (ref = No)**								
Yes	1.68 [0.97, 2.91]	*p* = 0.06	1.26 [0.70, 2.27]	*p* = 0.44	2.71^***^ [1.73, 4.25]	*p* = 0.00	1.51 [0.85, 2.70]	*p* = 0.16
N	467		467		467		467	

*Model 1, Unadjusted; Model 2, Adjusted for age, sex, and race; Model 3, Adjusted for age, sex, race, body mass index, diabetes, and hypertension*.

*** *p < 0.001*;

** *p < 0.01*;

* *p < 0.05*.

In secondary analyses among only people with myocardial infarction (MI) and subsequent analyses stratifying by presence or absence of pre-existing MI diagnosis, the results were not significantly different than those from when we stratified by the more inclusive CHD definition, with no statistically significant association between SVI and any pathologic outcome among patients with or without clinical MI (Supplemental Tables 1A,B).

## Discussion

In this investigation, we had the unique opportunity to evaluate associations of geospatially-derived social vulnerability with cardiac tissue-level pathology observed at autopsy. The primary significant association we observed was that, among individuals without known clinical HF, a higher proxy-marker of social vulnerability (SVI) was associated with a significantly higher adjusted odds of having myocardial fibrosis at autopsy: every 10% increase in SVI was associated with an approximately 13.7% increase in odds of having myocardial fibrosis at autopsy in these individuals without known HF.

Taken together, our findings corroborate prior data implicating SVI in subclinical – but potentially clinically relevant – myocardial tissue-level damage. For instance, Fibroblast Growth Factor 23 (FGF23), a key mediator of salt homeostasis shown to be elevated in high SVI communities and among individuals with high inorganic phosphate intake, has been established as a driver of myocardial fibrosis (18, 19). These phosphates may further disturb salt homeostasis, directly trigger pro-fibrotic fibroblast activation (20, 21), exacerbating vascular calcification (22). Epidemiologically, neighborhoods with better access to food resources in urban settings have lower premature CVD mortality (23), these healthy food sources tend to be less available for Black populations, and low availability of healthy foods is associated with a lower quality diet (24, 25). Therefore, it is plausible that SVI-associated neighborhood and environmental factors which contribute a high-salt, low quality diet ultimately drive both clinical and subclinical myocardial fibrosis.

Several other explanations for the association between SVI and myocardial fibrosis among patients without HF also exist. Individuals living in areas with high SVI may have diminished opportunity to pursue exercise, worsening their cardiovascular health and accelerating myocardial fibrosis (26). Factors that have previously been associated with increased physical activity, including environment walkability, safety from crime, walk-friendly infrastructure, and access to recreational facilities as well as public transport (27), may be absent in areas with SVI, furthering the link between SVI and myocardial fibrosis. Chronic psychosocial stress has previously been associated with cardiomyocyte dysfunction (28) and myocardial fibrosis in mice; of note, this pathological change occurred in the face of apparent adaptation (29). Areas with high SVI may place chronic stress on residents, furthering the development of myocardial fibrosis. Finally, patients living in areas of high SVI may experience barriers to healthcare access; patients experiencing high social deprivation have previously been found to have poor access to healthcare (30). As such, it is certainly possible that patients with high SVI who are systematically less likely to access care have a higher likelihood of having undiagnosed HF at death/autopsy, despite truly having myocardial fibrosis and HF.

Our study had several limitations which were largely unavoidable due to the nature of the cohort. Another limitation of this investigation is its focus on Illinois alone; this is due to limitations in the calculation of SVI, which is calculated at either the state or national-level. As the majority of patients were within Illinois state boundaries, patients outside of Illinois were excluded. Replication of this approach using larger, national cohorts could allow for the investigation of the association of SVI with pathologic traits on cardiac autopsy at the national level. Although our geographic region from the state of Illinois was large and racially/ethnically diverse, its findings may be skewed toward the characteristics of the Midwest. Of note, dietary patterns in the Midwest have previously been found to be unhealthy, and may be enriched in salt and/or phosphates (31–33). Among the cohort, a statistically significant association between SVI and myocardial fibrosis was found only in the absence of clinical heart failure, It may be the case that, after development of overt disease, traditional risk factors are more dominant in influencing tissue manifestations of cardiovascular disease. As such, among patients without heart failure, social vulnerability may be an important, actionable determinant of health. Myocardial fibrosis, even subclinically, has significant deleterious impacts on health, including greater susceptibility to arrhythmias (34), decreased exercise tolerance (35), and increased likelihood of sudden cardiac death (36). Broadly, improved access to the components of this metric may decrease social vulnerability and in turn mitigate the impact of myocardial fibrosis. A limitation of our investigation is that myocardial fibrosis was ascertained using a practice of presence/absence confirmation alone. As such, degree of myocardial fibrosis was not assessed, preventing direct quantification of the relationship between increasing SVI and extent of myocardial fibrosis. This would be interesting to investigate in future studies and potentially link to any preceding imaging tissue characterization data if available. Additionally, causes of death among participants were not heavily accounted for among the sample, and potential bias in death certificate may arise based on where the certificate was filed. It is also possible that differential medication availability and access by SVI status may differentially impact risk of developing cardiac pathology as observed at autopsy. Additionally, degree of myocardial fibrosis was not assessed, preventing direct quantification of the relationship between increasing SVI and extent of myocardial fibrosis. This would be interesting to investigate in future studies and potentially link to any preceding imaging tissue characterization data if available. Another important limitation in this study is the limited availability of data on prior hospitalizations, especially at out-of-network medical centers. This may have differentially impacted available data on HF diagnoses, such that persons with less in-system care may have been less likely to have a condition such as HF formally diagnosed even if they had true HF pathophysiology. This is especially true prior investigations that have established SVI and a risk factor for heart failure readmissions (37). An important limitation in our study is the reliance on adult clinical and geographic information. Prior investigations have noted that exposure to social stress in early childhood and developmental challenges increase the likelihood of cardiovascular events (38, 39). Future investigation into the comparative impact of childhood and adult social vulnerability would be of interest.

Despite these limitations, this analysis provided a unique opportunity to examine associations of geospatial proxies of social vulnerability with tissue-level pathology confirmed at autopsy. Our observation of a significant association of higher social vulnerability with myocardial fibrosis among people without known clinical HF merits replication in future studies linking clinical and pathology data; if confirmed, continued health and policy interventions targeting ways to mitigate onset and worsening of myocardial fibrosis in socially vulnerable communities are needed.

## Data Availability Statement

The raw data supporting the conclusions of this article will be made available by the authors, without undue resrvation.

## Author Contributions

AS and MF designed the research study and wrote the manuscript. AS and AR performed the analyses. MG, SK, JM, JO'N, and JL generated the dataset and assisted with analyses. All authors contributed to the interpretation of data and critical revision and editing of the manuscript.

## Funding

This work was funded by the American Heart Association Fellow-to-Faculty Award (16FTF31200010; PI: MF) and the National Institutes of Health R01HL156792 (PI: MF) and UL1TR001422 (PI: D'Aquila).

## Conflict of Interest

The authors declare that the research was conducted in the absence of any commercial or financial relationships that could be construed as a potential conflict of interest.

## Publisher's Note

All claims expressed in this article are solely those of the authors and do not necessarily represent those of their affiliated organizations, or those of the publisher, the editors and the reviewers. Any product that may be evaluated in this article, or claim that may be made by its manufacturer, is not guaranteed or endorsed by the publisher.
