# Supplementary material for: Associations of Social Vulnerability Index With Pathologic Myocardial Findings at Autopsy
Source: Front Cardiovasc Med. 2021 Dec 23;8:805278. doi: 10.3389/fcvm.2021.805278 (PMC8733155; doi:10.3389/fcvm.2021.805278)
Supplement: Supplementary file 1 [file Data_Sheet_1.PDF]

1.1 Supplemental Table 1A. Associations of Social Vulnerability Index with Cardiac Pathology at Autopsy Among Individuals with Clinical Diagnoses of Myocardial Infarction

	Coronary Atherosclerosis (Any)		Coronary Atherosclerosis (Severe)		Myocardial Fibrosis		Myocarditis and/or Pericardial Inflammation	
Predictor	OR 95% CI	P value	OR 95% CI	P value	OR 95% CI	P value	OR 95% CI	P value
Social Vulnerability Index (Model 1)	0.43 [0.08, 2.36]	p = 0.33	0.60 [0.22, 1.60]	p = 0.30	2.97 [0.94, 9.32]	p = 0.06	0.86 [0.27, 2.73]	p = 0.79
Social Vulnerability Index (Model 2)	0.55 [0.06, 4.97]	p = 0.60	1.12 [0.34, 3.69]	p = 0.85	3.13 [0.80, 12.23]	p = 0.10	0.83 [0.21, 3.28]	p = 0.79
Social Vulnerability Index (Model 3)	0.45 [0.04, 4.79]	p = 0.51	1.11 [0.33, 3.69]	p = 0.87	3.20 [0.81, 12.62]	p = 0.10	0.82 [0.20, 3.33]	p = 0.78
Race (ref = White)								
Asian/Hispanic/Other	0.90 [0.20, 4.03]	p = 0.89	0.52 [0.23, 1.15]	p = 0.11	0.94 [0.39, 2.23]	p = 0.88	1.30 [0.53, 3.19]	p = 0.57
Black	2.52 [0.51, 12.39]	p = 0.25	0.47 [0.21, 1.04]	p = 0.06	1.16 [0.48, 2.82]	p = 0.74	0.82 [0.32, 2.12]	p = 0.68
Sex (ref = Female)								
Male	2.89 [0.88, 9.52]	p = 0.08	1.01 [0.54, 1.88]	p = 0.98	1.67 [0.84, 3.30]	p = 0.14	1.26 [0.61, 2.62]	p = 0.53
Age	1.11 *** [1.06, 1.17]	p = 0.00	1.02 [1.00, 1.04]	p = 0.05	1.00 [0.98, 1.03]	p = 0.77	0.98 [0.95, 1.00]	p = 0.09
Diabetes Status (ref = No)								
Yes	2.50 [0.73, 8.58]	p = 0.14	1.71 [0.91, 3.20]	p = 0.09	1.04 [0.52, 2.06]	p = 0.92	1.29 [0.62, 2.67]	p = 0.49

Hypertension Status (ref = No)								
Yes	3.08 [0.84, 11.29]	p = 0.09	1.35 [0.73, 2.49]	p = 0.34	0.77 [0.39, 1.52]	p = 0.45	0.61 [0.30, 1.25]	p = 0.18
Body Mass Index	0.99 [0.92, 1.06]	p = 0.73	1.00 [0.96, 1.04]	p = 0.93	0.99 [0.94, 1.03]	p = 0.53	1.02 [0.98, 1.07]	p = 0.31
N	204		204		204		204	

Model 1: Unadjusted; Model 2: Adjusted for age, sex, and race; Model 3: Adjusted for age, sex, race, diabetes, hypertension, and body mass index; *** p < 0.001; ** p < 0.01; * p < 0.05.

1.2 Supplemental Table 1B. Associations of Social Vulnerability Index with Cardiac Pathology at Autopsy Among Individuals without Clinical Diagnoses of Myocardial Infarction

	Coronary Atherosclerosis (Any)		Coronary Atherosclerosis (Severe)		Myocardial Fibrosis		Myocarditis and/or Pericardial Inflammation	
Predictor	OR 95% CI	P value	OR 95% CI	P value	OR 95% CI	P value	OR 95% CI	P value
Social Vulnerability Index (Model 1)	0.74 [0.42, 1.31]	p = 0.30	1.15 [0.62, 2.13]	p = 0.65	1.41 [0.83, 2.40]	p = 0.21	0.98 [0.47, 2.06]	p = 0.96
Social Vulnerability Index (Model 2)	1.81 [0.89, 3.69]	p = 0.10	1.79 [0.87, 3.71]	p = 0.12	1.41 [0.77, 2.58]	p = 0.26	0.93 [0.41, 2.12]	p = 0.87
Social Vulnerability Index (Model 3)	1.63 [0.79, 3.37]	p = 0.19	1.71 [0.82, 3.55]	p = 0.15	1.31 [0.71, 2.42]	p = 0.39	0.90 [0.40, 2.05]	p = 0.81
Race (ref = White)								
Asian/Hispanic/Other	0.71 [0.43, 1.18]	p = 0.19	0.81 [0.49, 1.35]	p = 0.42	1.09 [0.71, 1.66]	p = 0.70	0.94 [0.53, 1.66]	p = 0.83
Black	0.56 * [0.34, 0.93]	p = 0.02	0.96 [0.58, 1.59]	p = 0.89	1.29 [0.84, 1.97]	p = 0.24	0.87 [0.49, 1.55]	p = 0.64
Sex (ref = Female)								

Male	1.47 [0.98, 2.19]	p = 0.06	1.71 ** [1.16, 2.52]	p = 0.01	1.30 [0.94, 1.81]	p = 0.12	1.16 [0.74, 1.81]	p = 0.52
Age	1.08 *** [1.06, 1.09]	p = 0.00	1.05 *** [1.03, 1.06]	p = 0.00	1.02 *** [1.01, 1.03]	p = 0.00	0.98 * [0.97, 1.00]	p = 0.03
Diabetes Status (ref = No)								
Yes	1.98 ** [1.28, 3.04]	p = 0.00	1.39 [0.93, 2.08]	p = 0.10	1.37 [0.97, 1.93]	p = 0.07	1.09 [0.68, 1.74]	p = 0.71
Hypertension Status (ref = No)								
Yes	1.18 [0.75, 1.84]	p = 0.47	0.96 [0.64, 1.44]	p = 0.84	1.47 * [1.04, 2.09]	p = 0.03	1.43 [0.88, 2.31]	p = 0.15
Body Mass Index	1.02 [0.99, 1.05]	p = 0.16	1.01 [0.99, 1.04]	p = 0.26	1.02 [1.00, 1.04]	p = 0.06	1.00 [0.97, 1.03]	p = 0.87
N	649		649		649		649	

Model 1: Unadjusted; Model 2: Adjusted for age, sex, and race; Model 3: Adjusted for age, sex, race, diabetes, hypertension, and body mass index; *** p < 0.001; ** p < 0.01; * p < 0.05.
